# Professor Miller’s “Micro-Organisms of the Human Mouth”

**Published:** 1890-12

**Authors:** 


### PROFESSOR MILLER'S "MICRO-ORGANISMS OF THE HUMAN MOUTH."

We have received from the publishers, S. S. White Dental Manufacturing Company, a copy of this anxiously-looked-for work of Professor Miller. The present English edition is, in many respects, superior to the German, and we deem it especially worthy of this special notice in advance of the general review which will appear in the January issue.

